# Dexmedetomidine sedation for transesophageal echocardiography during percutaneous atrial septal defect closure in adult

**DOI:** 10.12669/pjms.296.3616

**Published:** 2013

**Authors:** Jae Wook Jung, Gwang Cheol Go, Sang Yoon Jeon, Sira Bang, Ki Hwa Lee, Yong Han Kim, Dong-Kie Kim

**Affiliations:** 1 Jae Wook Jung, MD, Department of Anesthesiology and Pain Medicine, Haeundae Paik Hospital, Inje University, Busan, South Korea.; 2 Gwang Cheol Go, MD, Department of Anesthesiology and Pain Medicine, Haeundae Paik Hospital, Inje University, Busan, South Korea.; 3 Sang Yoon Jeon, MD, Department of Anesthesiology and Pain Medicine, Haeundae Paik Hospital, Inje University, Busan, South Korea.; 4 Sira Bang, MD, PhD, Department of Anesthesiology and Pain Medicine, Haeundae Paik Hospital, Inje University, Busan, South Korea.; 5 Ki Hwa Lee, MD, Department of Anesthesiology and Pain Medicine, Haeundae Paik Hospital, Inje University, Busan, South Korea.; 6 Yong Han Kim, MD, Department of Anesthesiology and Pain Medicine, Haeundae Paik Hospital, Inje University, Busan, South Korea.; 7 Dong-Kie Kim, MD, Department of Cardiology, Haeundae Paik Hospital, Inje University, Busan, South Korea.

**Keywords:** Atrial septal defect, Transesophageal echocardiography, Dexmedetomidine sedation

## Abstract

Atrial septal defect (ASD) is second common congenital heart disease that often leads to adult period. Intracardiac or transesophageal echocardiography (TEE) is essential for percutaneous closure of ASD using Amplatzer septal occluder. Dexmedetomidine (DEX), which is a highly selective α_2_-agonist, has sedative and analgesic properties without respiratory depression in the clinical dose range. We report percutaneous closure of ASD with TEE under DEX sedation.

## INTRODUCTION

Atrial septal defect (ASD) is the most frequent type of congenital heart disease.^1^ Successful closure of ASD was conducted by surgical procedure or transcutaneous cathether-placed device.^2^ Percutaneous method is preferred recently due to less invasive property.

Intracardiac or transesophageal echocardiography is indispensible for appropriate positioning and residual shunting of septal occluder device.^3^ Transesophageal echocardiography (TEE) has invasive property that is necessary for general anesthesia or deep sedation.^4^

Dexmedetomidine (DEX), which is recently, introduced α_2_-agonist that is more selective than clonidine, has been reported to be a useful sedative for diagnostic TEE.^5^ Dex produces sedative, analgesic, and anxiolytic effects without respiratory depression. We report a case of transcutaneous ASD closure under DEX sedation using TEE without intubation in adult patient.

## CASE REPORT

A 23-year-old female (height: 156.4 cm, weight: 50.6 kg) presented with chest discomfort and dyspnea in stressful situation. She had a transthoracic echocardiography (TTE) which found an ostium secondum ASD sized 1.0 cm with left to right shunt (Qp/Qs=1.6). Furthermore, mild tricuspid regurgitation and pulmonary regurgitation was revealed in preoperative TTE.

Percutaneous ASD closure was planned using fluoroscopy and intracardiac echocardiography (ICE) via femoral vein under local anesthesia. The patient was premedicated with diazepam 2 mg PO one hour before operation by cardiologist. Electrocardiography, pulse oximeter and noninvasive blood pressure were measured in 5 minutes interval. After puncture of right femoral vein with topical injection of 2% lidocaine, operator found that ICE had some trouble. Cardiologist requested emergent consultation to anesthesiologist with patient’s consent. Medical staff decided to perform ASD closure using TEE. General anesthesia deserved consideration because TEE has more invasive property than ICE. However, the patient refused both general anesthesia with intubation and delay of procedure. Anesthesiologist decided to sedate her using dexmedetomidine for TEE.

**Fig.1:**
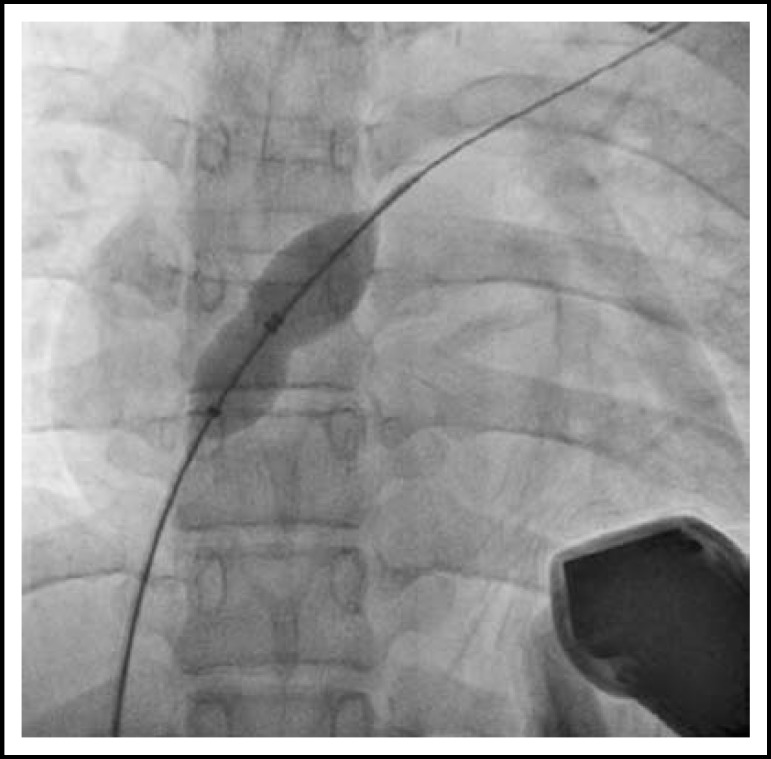
Balloon sizing of atrial septal defect under fluoroscopy

**Fig.2:**
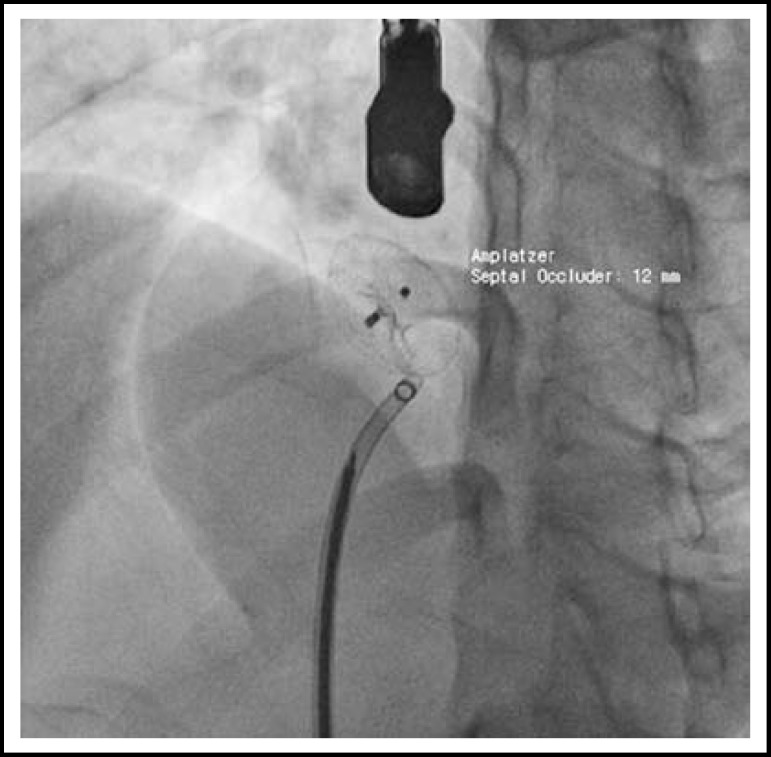
Fluroscopy represented that Amplatzer septal occluder was positioned in ASD

Bispectral Index (BIS) was monitored as smoothing time 15 seconds in addition. Dexmedetomidine (Precedex^®^, Hospira, USA) was started 1 ㎍/kg over 10 minutes and followed by 0.2 ㎍/kg/hr. Blood pressure was 134/72 mmHg, and heart rate (HR) was 94 beats per minute (bpm). The patient maintained spontaneous breathing using a nasal cannula with 100% oxygen of 3 L/min flow. After 15 minutes of infusion, BIS value was near 60. BIS Signal Quality Index (BIS-SQI) and electromyography (BIS-EMG) was 100 and 30, respectively. Ramsay scale was level 5 that patient responding sluggishly to a light glabellar tap or to verbal stimulus. Unfractionated heparin 6,000 IU was injected on intravenous route. Multipurpose catheter was inserted on femoral vein, and guide-wire was passed through ASD under TEE. Just before insertion of TEE, blood pressure was 96/57 mmHg and HR was 72 bpm. BIS value was raised to 70 after TEE insertion. BIS-SQI and BIS-EMG was changed to about 80 and 40, respectively. HR was raised to 87 bpm. Ramsay scale was level 3 that respond to commands only. The size of ASD was measured by size balloon via guide-wire (Fig.1). Amplatzer septal occluder (12mm) was positioned by using device delivery catheter in optimal location, and residual shunting was not visible by TEE (Fig.2). The patient was moved to recovery unit after completion of skin suture. Thirty minutes was consumed for total procedure. After one hour stay of recovery unit, she was admitted to general ward.

At the night of operation day, blood pressure was dropped to 70/40. The dopamine was infused as 5 ㎍/kg/min and fluid was increased. BP was maintained 90/50 after discontinue of dopamine in next day. Postoperative TTE represented no shunt flow and mild pulmonary regurgitation. The patient was discharged without any symptom or complication on seventh postoperative day.

## DISCUSSION

Ostium secundum type of ASD is most frequent in all ASD. Open heart surgery with sternotomy was considered as standard treatment over the past several decades. Since percutaneous ASD closure was first performed by King and Mills, transcutaneous repair has become a well-established and alternative method in children and adults.^6^ Cautious monitoring is necessary for several complications like arrhythmia or device embolism. Quek et al.^7^ reported that minimal invasive closure of ASD has some advantages compared to conventional surgical repair – less morbidity and short hospital stay. However, there was no cost saving due to high cost of occlusive device in Singapore. In over 60 years group, percutaneous occlusion of ASD has benefit like symptom reduction, improvement of functional exercise capacity, and positive right-heart remodeling after three month follow up.^8^

General anesthesia with endotracheal intubation is essential for minimal invasive ASD closure in children because of TEE insertion.^4^ In adult patient, TEE can be applied with proper sedative like DEX without intubation.^5^ Transcutaneous ASD closure could be performed using ICE under injection of local anesthetics. However, the patience is necessary for TEE according to circumstances.

Respiratory depression is very dangerous during TEE because it is difficult to ventilate with mask manually. Most of intravenous anesthetics have a potential risk of apnea with the exception of DEX and ketamine.^9^ DEX is highly selective α_2_-adrenergic receptor agonist which is a useful sedative without respiratory depression and has an analgesic property.^10^ DEX is 7 to 8 times more specific to α_2 _than α_1_-receptor.^11^ DEX maintains stable cardiovascular function compared to other sedatives. Hypertension can be caused as well by rapid or large concentration infusion, especially initial time that is short hypertensive phase. This patient represents stable BP, HR and SpO_2_. Emergency airway kit including laryngoscope and endotracheal tube has to be prepared for respiratory problem.

Ramsay sedation scale is widely used method to define level of sedation divided into six grades. Observers have a possibility to interpret as different scale because applying stimulus and evaluating responses are not objective. Furthermore, the patient with TEE is not simple to assess the level of sedation. To overcome cumbersome problems like these, BIS was applied to this patient.

## CONCLUSION

This report presents the successful closure of ASD by percutaneous invasive method using Amplatzer septal device without intubation in adult patient. Sedation using dexmedetomidine is effective in transcutaneous ASD closure with Transesophageal echocardiography.
